# Trends in overweight and obesity in children under 24 months of age in Mexico (2012-2020): analysis of four national health surveys

**DOI:** 10.1590/0102-311XEN046123

**Published:** 2023-12-22

**Authors:** Velia Margarita Cárdenas-Villarreal, Lucia Hernandez-Barrera, Danilo Castro-Sifuentes, Milton Carlos Guevara-Valtier, Belem Trejo-Valdivia

**Affiliations:** 1 Facultad de Enfermería, Universidad Autónoma de Nuevo León, Monterrey, México.; 2 Instituto Nacional de Salud Pública, Cuernavaca, México.

**Keywords:** Infants, Overweight, Obesity, Population, Surveys, Lactantes, Sobrepeso, Obesidad, Población, Encuestas, Lactentes, Sobrepeso, Obesidade, População, Inquéritos

## Abstract

The prevalence of childhood obesity has increased rapidly in Mexico, with significant consequences for the population’s health in the future. Little is known about the prevalence of obesity in children under two years of age, even though this life stage is fundamental to prevent this condition. This study aims to determine the magnitude, distribution, and trends of overweight and obesity in children under 24 months of age using the *Mexican National Health and Nutrition Surveys* (ENSANUT) conducted in the last 10 years. The data presented here are derived from four ENSANUTs, carried out in Mexico in 2012, 2016, 2018, and 2020. They include 6,719 infants under 24 months with complete anthropometric data (weight/height) by age, gender, Indigeneity, area of residence, and socioeconomic status. The risk of overweight levels and overweight + obesity rates were calculated according to World Health Organization guidelines. We identified that infants < 12 months currently have a higher prevalence of overweight + obesity (10.3%) and that those aged 12 to 23 months are generally at a higher risk of overweight (26.1%). The most relevant findings of this study, linking weight trends to sex, region, socioeconomic status, and indigeneity, show that overweight and obesity prevalences vary across the Mexican population, without presenting a specific behavior. There is a high prevalence of overweight and obesity among Mexican infants and a slight trend toward increased obesity in infants < 12 months. Weight monitoring and obesity prevention interventions focused on the first 1,000 days of life are essential.

## Resumen

La prevalencia de la obesidad infantil ha aumentado rápidamente en México, y tiene consecuencias para la salud de la población en el futuro. Poco se sabe sobre la prevalencia de obesidad en niños que tienen menos de dos años, etapa fundamental para la prevención de esta condición. El objetivo fue determinar la magnitud, la distribución y las tendencias de sobrepeso y obesidad en niños menores de 24 meses de edad utilizando las *Encuestas Nacionales de Salud y Nutrición* (ENSANUT) en México en los últimos 10 años. Los datos presentados se derivan de cuatro ENSANUTs en México: 2012, 2016, 2018 y 2020. Los datos incluyen 6.719 menores de 24 meses de edad con datos antropométricos completos (peso/altura) por edad, sexo, etnicidad, lugar de residencia y nivel socioeconómico. Se calculó el riesgo de sobrepeso y sobrepeso + obesidad siguiendo las directrices de la Organización Mundial de la Salud. Se identificó que los menores de 12 meses tienen una prevalencia más alta de sobrepeso + obesidad (10,3%), y los que tienen entre 12 y 23 meses tienen riesgo de sobrepeso más alto (26,1%). Los hallazgos más relevantes por sexo, región, nivel socioeconómico y etnicidad de este estudio que las prevalencias de sobrepeso y obesidad varían en la población mexicana, sin presentar un comportamiento específico. Hay una alta prevalencia de sobrepeso y obesidad en niños mexicanos, y una ligera tendencia de aumento de obesidad en menores de 12 meses. El monitoreo del peso y las intervenciones de prevención de obesidad centradas en los primeros 1.000 días de vida son esenciales.

## Resumo

A prevalência da obesidade infantil tem aumentado rapidamente no México, com consequências para a saúde da população no futuro. Pouco se sabe sobre a prevalência de obesidade em crianças menores de dois anos de idade, etapa fundamental para a prevenção desta condição. Nosso objetivo foi determinar a magnitude, distribuição e tendências de sobrepeso e obesidade em crianças menores de 24 meses de idade usando as *Pesquisas Nacionais de Saúde e Nutrição* (ENSANUT) no México nos últimos 10 anos. Os dados apresentados são derivados de quatro ENSANUTs no México: 2012, 2016, 2018 e 2020. Os dados incluem 6.719 crianças menores de 24 meses e seus dados antropométricos completos (peso/altura) por idade, sexo, etnicidade, área de residência e nível socioeconômico. O risco de sobrepeso e sobrepeso + obesidade foram calculados seguindo as diretrizes da Organização Mundial da Saúde. Foi identificado que crianças menores de 12 meses atualmente têm uma prevalência maior de sobrepeso + obesidade (10,3%), e aqueles com idade de 12 a 23 meses têm mais risco de sobrepeso (26,1%). Os achados mais relevantes por sexo, região, nível socioeconômico e etnicidade deste estudo mostram um México heterogêneo em relação a sobrepeso e obesidade sem apresentar um comportamento específico. Há uma alta prevalência de sobrepeso e obesidade em bebês mexicanos, e uma leve tendência de aumento da obesidade em crianças menores de 12 meses. O monitoramento do peso e as intervenções de prevenção de obesidade focadas nos primeiros 1.000 dias de vida são essenciais.

## Introduction

Overweight and obesity currently affects the health of children worldwide, and its prevalence continues to increase ^1^. The estimate for the prevalence of overweight and obesity in children under 5 years of age may vary in different countries, regions, and according to the definition with which it is measured and the estimated global prevalence for this condition has been estimated to be 4.8% in 1990 and increased to 5.9% in 2018 ^2^ ^,^ ^3^ ^,^ ^4^.

Overweight and obesity in early childhood has become a key factor for health decision-makers, because overweight and obesity has been associated with the increase in pediatric diseases that were previously considered diseases of adulthood ^5^ ^,^ ^6^. These include noncommunicable diseases such as type 2 diabetes, hypertension, depression ^7^, psychosocial problems ^6^, and the risk of premature death in middle age ^8^ ^,^ ^9^. In addition, individuals who develop obesity in the first year of life have been found to have a greater predisposition to obesity during all life stages ^10^. Hence, it is important to prevent and control obesity at an earlier age.

International health organizations have made efforts to formulate public health policies focused on the prevention and treatment of childhood obesity. For this process, they considered the first 1,000 days of life - the period from conception to the 24th month of life - as the best time to prevent overweight and obesity and its adverse consequences, given that this period is the most important for the development of children and the formation of eating habits, and for the establishment of healthy habits that could last a lifetime ^11^ ^,^ ^12^ ^,^ ^13^.

Like other countries, Mexico has been immersed in the overweight and obesity epidemic that affects millions of infants. The *Mexican National Health and Nutrition Surveys* (ENSANUT; *Encuesta Nacional de Salud y Nutrición*) have documented the progression of overweight and obesity prevalence in all population groups in Mexico during the past three decades, and found that this condition is increasingly affecting children. From these data, we can highlight the 6-fold raise in overweight and obesity prevalence revealed by ENSANUT 2016, with rates increasing from 5.3% in children under 5 years of age to 31.4% in schoolchildren aged 5-11 years, and the 4.3-fold increase in overweight and obesity prevalence found by ENSANUT 2018, with rates going from 8.2% in children aged 0-4 years to 35.6% in schoolchildren ^14^. This epidemiological information establishes that overweight and obesity prevalence in children under 11 years of age is almost half of the peak prevalence observed in the adult population, a worrying percentage for children’s health.

There is evidence that the first years of life are essential for laying the future foundations of well-being and improving health conditions throughout life ^15^. Therefore, the preventive actions carried out during this period reduce future risks of malnutrition, micronutrient deficiency, and obesity ^16^.

To date, no specific information has been reported on the trend in overweight + obesity prevalence in children under two years of age. This information could make it possible to monitor the trajectory of this condition and contribute to decision-making for public preventive policies in the first two years of life that aim to stop or revert what has been called the obesity epidemic in this population ^17^. Therefore, this study aims to determine the prevalence of overweight and obesity in children < 24-months old in Mexico and to compare estimates and trends considering the ENSANUTs conducted in 2012, 2016, 2018-2019, and 2020.

## Materials and methods

The information from the ENSANUTs conducted in 2012, 2016, 2018-2019, and 2020 was analyzed. These surveys are nationally representative and comparable in their methodological design - a probabilistic, stratified, cluster design. The 2012 ^18^ and 2018-2019 ^19^ surveys are representative at national, regional, and state levels. The 2016 ^20^ survey is nationally and regionally representative. Annual surveys have been carried out since 2020, maintaining representativeness at the national and regional levels, but not at the state level, which means the sample size is smaller in these surveys. The ENSANUT sampling procedures, databases, and questionnaires are publicly available and can be accessed at the website https://ensanut.insp.mx/index.php. This study included only children < 24 months of age, although more age groups are included in the ENSANUT.

### Study variables

To assess the nutritional status of the infants, trained personnel performed anthropometric measurements from all the children using internationally established standardized protocols ^21^ ^,^ ^22^. Body weight, in kilograms, was measured with a Seca electronic scale (https://www.seca.com/es_mx.html), and height/length, in centimeters, was measured with a Seca wall stadiometer.

The anthropometry of the children was processed from the data on their weight and height or length by age and sex, provided by the ENSANUT, using the macro of the World Health Organization (WHO) growth standards ^13^.

The Z-score of the body mass index (BMI) for age was also calculated using the aforementioned data. The risk of overweight was classified as a Z-BMI score > 1 standard deviation and ≤ 2 standard deviations. Overweight + obesity was defined by a Z-score +2 times the standard deviation of the reference mean. Data marked as biologically implausible according to WHO guidelines were defined by weight-for-length Z < 5 or > 5 ^23^.

### Covariates

(i) Area of residence: the children’s areas of residence were classified depending on the number of inhabitants: they could be urban (2,500 or more inhabitants) or rural (less than 2,500 inhabitants).

(ii) Region: for this study, the Mexican territory was divided into four regions: North (Baja California, Baja California Sur, Chihuahua, Coahuila, Durango, Nuevo Leon, Sonora, and Tamaulipas); Central (Aguascalientes, Colima, Guanajuato, Jalisco, Michoacan, Morelos, Nayarit, Queretaro, Sinaloa, and Zacatecas); Mexico City and State of Mexico; and South (Campeche, Chiapas, Guerrero, Hidalgo, Puebla, Oaxaca, Quintana Roo, Tlaxcala, Tabasco, Veracruz, and Yucatan).

(iii) Indigeneity: the element of indigeneity was defined as present if any member of the child’s family spoke an Indigenous language.

(iv) Socioeconomic status: this index was constructed by analyzing the main components along with information on the children’s home (e.g., number of rooms and bathrooms, availability of running water, and building materials), as well as their household goods (e.g., washing machine, microwave, stove, television, etc.). The index was divided into tertiles and categorized as low, medium, and high ^19^.

### Statistical analysis

This study presents a descriptive analysis of the current prevalence of nutritional status and a comparison between the different nutritional status categories in the four surveys over time. For overweitgh + obesity, the results suggest different behaviors by age group in children aged < 12 months and in those aged between 11-23 months. We therefore considered analyzing them separately.

For overweight and obesity prevalence alone, comparisons were made by type of area and region of residence. The analysis was carried out with the statistical program Stata v.14 (https://www.stata.com), using the *svy* module for complex samples.

### Ethical aspects

The Research Ethics Committee and the Biosafety Committee (Mexican National Institute of Public Health), in compliance with the written informed consent of the participants, approved the ENSANUTs. This study preserves participants’ anonymity.

## Results

We analyzed information on 6,719 children < 24 months of age from the four ENSANUTs. The following sample sizes were used in each year’s survey: 3,854 in 2012; 1,096 in 2016; 1,234 in 2018-2019; and 535 in 2020. Each sample was analyzed according to the age group of the infants (< 12 months and 12-23 months). Table 1 shows the characteristics of each study sample by age group. The distribution of characteristics by sex, area, region, and indigeneity was similar among ENSANUTs, but the distribution of characteristics by socioeconomic status was not: in 2016, the proportion of participants with a high socioeconomic status was higher than in 2012, 2018-2019, and 2020.

**Table 1:** Sociodemographic characteristics of each sample by age group of children aged < 24 months. *Mexican National Health and Nutrition Survey* (ENSANUT), 2012-2020.

Characteristics	ENSANUT 2012			ENSANUT 2016			ENSANUT 2018-2019			ENSANUT 2020		
	n	N (thousands)	%	n	N (thousands)	%	n	N (thousands)	%	n	N (thousands)	%
Total	3,854	4,022.4		1,096	4,188.4		1,234	3,182.5		535	3,330.2	
< 12 months												
Subtotal	1,741	1,896.1		717	1,915.8		511	1,428.6		256	1,606.7	
Sex												
Male	852	935.7	49.3	166	1,050.2	54.8	246	728.3	51	134	810.2	50.4
Female	889	960.4	50.7	172	865.5	45.2	265	700.3	49	122	796.4	49.6
Area												
Urban	1,098	1,428.1	75.3	137	1,331.7	69.5	328	1,013.9	71	178	1,182.7	73.6
Rural	643	468.0	24.7	201	584.0	30.5	183	414.6	29	78	423.9	26.4
Region												
North	402	393.9	20.8	78	480.9	25.1	104	289.4	20.3	37	266.2	16.6
Center	587	527.8	27.8	104	613.7	32.0	182	454.3	31.8	95	495.5	30.8
Mexico City and State of Mexico	98	379.8	20.0	44	232.9	12.2	15	194.2	13.6	38	243.0	15.1
South	654	594.4	31.4	112	588.1	30.7	210	490.5	34.3	86	601.8	37.5
Socioeconomic status												
Low	685	622.5	32.8	152	567.5	29.6	212	566.3	39.6	104	616.3	38.4
Middle	627	676.8	35.7	99	680.4	35.5	175	478.1	33.5	90	605.2	37.7
High	429	596.8	31.5	87	667.8	34.9	124	384.1	26.9	62	385.1	24.0
Indigenous language												
No	1,507	1,705.0	89.9	288	1,775.5	92.7	448	1,295.6	90.1	226	1,414.0	88.0
Yes	234	191.1	10.1	50	140.2	7.3	63	132.9	9.3	30	192.7	12.0
12-23 months												
Subtotal	2,113	2,126.2		379	2,272.6		723	1,753.9		279	1,723.5	
Sex												
Male	1,054	1,112.4	52.3	189	1,085.9	47.8	384	867.0	49.4	160	977.0	56.7
Female	1,059	1,013.8	47.7	190	1,186.7	52.2	348	886.8	50.6	119	746.5	43.3
Area												
Urban	1,293	1,573.6	74.0	172	1,748.0	76.9	464	1,013.9	74.7	203	1,234.2	71.6
Rural	820	552.6	26.0	207	524.6	23.1	268	414.6	25.3	76	489.2	28.4
Region												
North	460	415.4	19.5	83	341.5	15.0	141	336.2	19.2	45	310.5	18.0
Central	705	581.1	27.3	90	637.3	28.0	257	517.1	29.5	91	481.3	27.9
Mexico City and State of Mexico	114	417.4	19.6	54	514.7	22.6	27	303.4	17.3	49	315.6	18.3
South	834	712.2	33.5	152	779.0	34.3	307	597.0	34	94	616.0	35.7
Socioeconomic status												
Low	857	715.3	33.6	159	604.2	26.6	317	637.3	36.3	130	761.9	44.2
Medium	729	732.8	34.5	126	598.7	26.3	244	675.3	38.5	98	602.8	35.0
High	527	678.1	31.9	94	1,069.7	47.1	171	441.2	25.2	51	358.8	20.8
Indigenous language												
No	1,806	1,904.1	89.6	321	1,998.3	87.9	634	1,558.6	88.9	248	1,508.0	87.5
Yes	307	222.1	10.4	58	274.3	12.1	98	195.3	11.1	31	215.5	12.5

**Figure 1:**
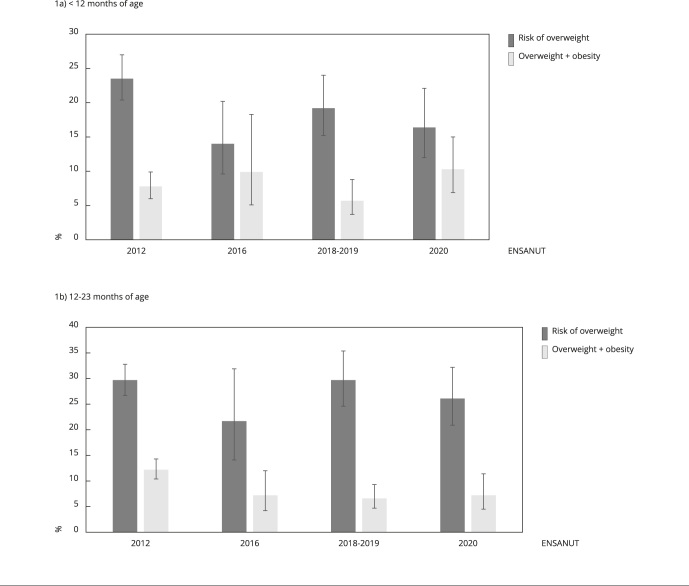
Comparison of the national prevalence of the risk of overweight and overweight + obesity in children aged < 24 months. *Mexican National Health and Nutrition Survey* (ENSANUT), 2012-2020.

Figure 1 shows the national prevalence of risk of overweight and overweight + obesity by age group and ENSANUT. When comparing the data distribution among the different surveys, we observed that both age groups have the same pattern. Overweight + obesity prevalence in infants aged < 12 months increased over time. In 2012, the estimate for this prevalence was 7.8% (95%CI: 6.0; 9.9), and in 2020, it was 10.3% (95%CI: 6.9; 15.0) - that is, it increased by 32%. In 2012 an overweight + obesity prevalence of 12.2% (95%CI: 10.4; 14.3) was observed among infants aged 12-23 months, but this rate subsequently decreased, remaining at 6.6% (95%CI: 4.7; 9.3) from 2018-2019 to 2020. A high risk of overweight prevalence was also observed in this age group, going from 29.7% (95%CI: 20.3; 27.4) in 2012 to 26.1% (95%CI: 20.9; 32.2) in 2020. The covariates by ENSANUT according to these age groups were subsequently analyzed considering the observed differences (Table 2).

**Table 2:** National prevalence of risk of overweight, overweight and obesity by sex, area, region, socioeconomic status, and Indigenous language for each age group of children aged < 24 months in Mexico and per *Mexican National Health and Nutrition Survey* (ENSANUT), 2012-2020.

Characteristics	ENSANUT 2012		ENSANUT 2016		ENSANUT 2018-2019		ENSANUT 2020	
	Risk of overweight [% (95%CI)]	Overweight + obesity * [% (95%CI)]	Risk of overweight [% (95%CI)]	Overweight + obesity * [% (95%CI)]	Risk of overweight [% (95%CI)]	Overweight + obesity * [% (95%CI)]	Risk of overweight [% (95%CI)]	Overweight + obesity * [% (95%CI)]
< 12 months of age								
Subtotal	23.5 (20.4; 27.0)	7.8 (6.0; 9.9)	14.0 (9.6; 20.2)	9.9 (5.1; 18.3)	19.2 (15.2;24.0)	5.7 (3.7; 8.8)	16.4 (12.0; 22.1)	10.3 (6.9; 15.0)
Sex								
Male	24.2 (19.9; 29.0)	8.0 (5.6; 11.2)	13.6 (8.3; 21.6)	14.5 (6.7; 28.8)	18.9 (13.1; 26.5)	9.0 (5.3; 14.9)	19.2 (12.8; 27.7)	11.9 (7.6; 18.2)
Female	22.9 (19.0; 27.4)	7.6 (5.6; 10.2)	14.4 (8.4; 23.7)	4.4 (1.6; 11.3)	19.5 (13.9; 26.7)	2.4 (1.2; 4.5)	13.6 (8.2; 21.6)	8.6 (4.2; 16.8)
Area								
Urban	23.7 (19.8; 28.1)	8.5 (6.4; 11.3)	10.7 (6.2; 18.0)	10.8 (4.7; 23.2)	19.0 (13.7; 25.0)	5.8 (3.4; 9.9)	16.0 (11.0; 22.8)	10.4 (6.7; 15.7)
Rural	23.1 (18.9; 27.8)	5.5 (3.7; 8.0)	21.6 (13.8; 32.0)	7.7 (3.4; 16.5)	19.7 (14.6; 26.1)	5.5 (2.7; 10.8)	17.4 (9.4; 30.0)	10.0 (4.1; 22.2)
Region								
North	29.2 (23.6; 35.4)	11.6 (7.6; 17.2)	14.0 (6.7; 26.8)	11.7 (3.1; 35.5)	28.6 (17.1; 43.7)	6.6 (2.6; 15.7)	19.3 (8.6; 37.6)	7.2 (1.7; 25.4)
Central	20.6 (16.2; 25.7)	8.2 (5.2; 12.8)	16.7 (8.2; 31.3)	10.0 (2.4; 33.8)	19.1 (13.0; 27.2)	7.8 (3.9; 15.0)	15.5 (8.9; 25.5)	4.7 (1.9; 11.3)
Mexico City and State of Mexico	26.4 (16.0; 40.4)	4.8 (1.8; 12.4)	10.1 (4.5; 21.3)	12.1 (3.6; 33.9)	5.9 (0.7; 36.9)	0.0 (0.0; 0.0)	15.6 (5.6; 36.3)	11.7 (3.3; 33.9)
South	20.5 (16.7; 25.0)	6.7 (4.5; 9.9)	12.7 (6.2; 24.3)	7.5 (2.7; 18.8)	19.0 (14.1; 25.3)	5.6 (2.8; 10.9)	16.2 (9.7; 25.8)	15.6 (9.9; 23.7)
Socioeconomic status								
Low	24.7 (19.4; 30.9)	7.6 (5.1; 11.1)	17.6 (10.7; 27.5)	6.1 (2.3; 15.0)	18.1 (12.6; 25.4)	3.1 (1.5; 6.1)	15.5 (8.8; 25.9)	14.9 (8.3; 25.3)
Middle	22.4 (17.8; 27.8)	9.0 (6.1; 13.1)	15.4 (7.4; 29.3)	2.9 (1.0; 8.4)	18.9 (11.8; 28.9)	8.0 (4.3; 14.3)	19.7 (11.8; 31.0)	8.1 (3.8; 16.1)
High	23.6 (17.5; 31.0)	6.6 (3.9; 11.0)	9.4 (4.3; 19.5)	20.6 (8.7; 41.3)	21.2 (12.7; 33.1)	6.9 (2.6; 16.8)	12.6 (6.5; 23.0)	6.3 (2.2; 16.7)
Indigenous language								
No	23.7 (20.3; 27.4)	7.9 (6.1; 10.3)	13.8 (9.2; 20.2)	10.6 (5.4; 19.8)	19.9 (15.4; 25.4)	6.0 (3.8; 9.3)	14.7 (10.3; 20.6)	9.5 (6.3; 14.1)
Yes	22.1 (15.3; 30.9)	6.2 (3.7; 10.4)	16.4 (5.1; 41.8)	0.6 (0.1; 4.7)	12.7 (6.7; 22.8)	3.5 (1.0; 11.1)	28.9 (14.7; 48.7)	15.7 (4.9; 39.9)
12-23 months of age								
Subtotal	29.7 (26.7; 32.8)	12.2 (10.4; 14.3)	21.7 (14.1; 31.9)	7.2 (4.2; 12.0)	29.7 (24.6; 35.4)	6.6 (4.7; 9.3)	26.1 (20.9; 32.2)	7.2 (4.5; 11.4)
Sex								
Male	30.3 (26.0; 34.8)	12.8 (10.2; 16.0)	18.7 (10.2; 31.8)	5.6 (2.9; 10.6)	34.3 (27.2; 42.1)	6.4 (3.9; 10.2)	25.0 (18.4; 33.1)	7.6 (4.1; 13.7)
Female	29.0 (24.8; 33.7)	11.6 (9.1; 14.6)	24.5 (13.1; 41.1)	8.7 (3.9; 18.3)	25.3 (18.3; 33.8)	6.9 (4.1; 11.4)	27.6 (9.3; 37.8)	6.7(3.2; 13.2)
Area								
Urban	29.6 (25.9; 33.6)	12.5 (10.3; 15.2)	22.4 (13.1; 35.8)	8.1 (4.3; 14.8)	31.8 (25.5; 38.8)	5.4 (3.4; 8.4)	27.2 (20.9; 34.7)	6.5 (3.7; 11.2)
Rural	29.9 (26.0; 34.2)	11.3 (8.4; 14.9)	19.3 (12.0; 29.3)	4.2 (2.3; 7.7)	23.7 (17.7; 31.0)	10.3 (6.0; 17.0)	23.3 (15.2; 34.0)	9.0 (4.0; 19.1)
Region								
North	29.1 (23.6; 35.3)	17.5 (12.9; 23.1)	28.2 (17.3; 42.6)	16.9 (6.0; 39.5)	30.2 (20.5; 42.1)	9.7 (5.1; 17.7)	28.3 (17.5; 42.3)	9.6 (3.9; 21.8)
Central	31.7 (26.5; 37.3)	14.3 (10.6; 18.9)	20.2 (8.1; 42.2)	1.9 (0.4; 9.0)	25.4 (18.5; 34.0)	7.4 (4.4; 12.3)	25.3 (16.9; 36.1)	8.7 (3.8; 18.7)
Mexico City and State of Mexico	34.4 (24.9; 45.2)	4.6 (2.0; 10.0)	29.7 (9.1; 64.0)	3.6 (1.0; 12.4)	40.1 (22.0; 61.4)	6.0 (1.5; 21.0)	30.5 (18.2; 46.5)	2.8 (0.7; 10.4)
South	25.6 (21.9; 29.7)	11.9 (9.4; 15.1)	14.5 (9.9; 20.7)	9.8 (5.5; 16.9)	27.9 (21.0; 36.0)	4.6 (2.8; 7.4)	23.5 (15.3; 34.2)	7.1 (3.1; 15.5)
Socioeconomic status								
Low	28.3 (23.7; 33.4)	10.5 (7.6; 14.3)	29.2 (13.1; 52.9)	5.0 (2.1; 11.4)	21.3 (15.2; 28.9)	5.9 (3.2; 10.9)	27.4 (19.8; 36.6)	8.6 (4.3; 16.4)
Medium	29.5 (24.9; 34.6)	13.4 (10.2; 17.3)	22.8 (13.5; 35.8)	14.9 (7.3; 28.0)	38.2 (29.3; 48.0)	4.6 (2.8; 7.6)	21.0 (13.2; 31.7)	8.3 (4.1; 16.2)
High	31.3 (24.8; 38.6)	12.8 (9.6; 16.9)	16.7 (8.4; 30.7)	4.0 (1.4; 10.8)	29.0 (19.5; 40.7)	10.7 (5.6; 19.5)	32.1 (18.8; 49.0)	2.5 (0.6; 9.8)
Indigenous language								
No	29.5 (26.3; 32.9)	12.1 (10.2; 14.4)	19.0 (12.8; 27.3)	7.9 (4.5; 13.4)	30.0 (24.6; 36.0)	7.0 (4.9; 10.0)	27.7 (22.1; 34.2)	6.9 (4.3; 10.9)
Yes	31.5 (25.5; 38.2)	13.0 (8.5; 19.2)	45.0 (13.2; 81.4)	1.3 (0.2; 6.5)	27.2 (17.4; 39.9)	3.7 (1.4; 9.7)	15.1 (5.7; 34.1)	9.8 (2.5; 31.5)

* Data are adjusted for the survey design. The prevalence of overweight + obesity includes the prevalence of overweight + the prevalence of obesity found in the survey and was assessed using the cut-off point of the World Health Organization growth standards ^13^.

### Infants < 12 months

Slight differences in trends by sex were observed over time (from 2012 to 2020): in boys, overweight and obesity prevalence increased more than (8.0%; 95%CI: 5.6; 11.2 vs. 11.9%; 95%CI: 7.6; 1.8) in girls (7.6%; 95%CI: 5.6; 10.2 vs. 8.6%; 95%CI: 4.2; 16.8). Trends by area of residence also differed: a higher overweight + obesity prevalence in 2020 was observed in the urban area (8.5%; 95%CI: 6.4; 11.3 vs. 10.4%; 95%CI: 6.7; 15.7), although it was not significantly different from the prevalence observed in the rural area (5.5%; 95%CI: 3.7; 8.0 vs. 10.0%; 95%CI: 4.1; 22.2). Overweight + obesity prevalence does not show a consistent pattern by region, possibly due to the sample size and distribution. The prevalence by socioeconomic status also did not show a consistent pattern; however, in individuals with a low socioeconomic status, the increase in overweight + obesity prevalence almost doubled, from 7.6% (95%CI: 5.1; 11.1) in 2012 to 14.9% (95%CI: 8.3; 25.3) in 2020. By indigeneity, overweight + obesity prevalence also did not show a consistent pattern.

### Infants aged 12-23 months

In this age group, no differences were observed for overweight + obesity prevalence by sex. However, this prevalence did differ by area of residence: overweight + obesity prevalences in the urban area were higher in 2012 (12.5%; 95%CI: 10.3; 15.2) and 2016 (8.1%; 95%CI: 4.3; 14.8), and, in the rural area, a higher overweight + obesity prevalence was observed in 2018-2019 (10.3%; 95%CI: 6.0; 17.0) and 2020 (9.0%; 95%CI: 4.0; 19.1). Overweight + obesity prevalences also differed by region: from 2012 to 2020, the prevalence in this age group was consistently higher in the Northern part of the country, and it was lower in the Mexico City-State of Mexico than in the Central and South regions. Moreover, overweight + obesity rates decreased slightly over time in the Central and South regions. A decrease in overweight + obesity prevalence was observed in individuals with a high socioeconomic status level, from 12.8% (95%CI: 9.6; 16.9) in 2012 to 2.5% (95%CI: 0.6; 9.8) in 2020.

By indigeneity, imprecise estimates were obtained in both age groups. Generally, only some individuals speak an Indigenous language, which generates inaccurate estimates of overweight + obesity indicators, so it is not surprising that a specific pattern has not yet been observed. Overall, a decrease in overweight + obesity prevalence was observed, from 12.1% (95%CI: 10.2; 14.4) in 2012 to 6.9% (95%CI: 4.3; 10.9) in 2020, particularly in those who do not speak an Indigenous language.

## Discussion

This study describes overweight + obesity prevalence in the population of infants aged under 24 months in Mexico, considering data from four ENSANUTs, carried out in 2012, 2016, 2018-2019, and 2020. It was observed that the prevalence of this condition in Mexican infants is high, as well as in children from other countries, such as the United States ^24^ ^,^ ^25^, according to the few studies that have documented information about this age group. Another interesting finding, although inconclusive, is that even though there is no evident trend toward an increased prevalence of overweight + obesity in this group, infants < 12 months of age had a higher rate of overweight + obesity, and those aged 12-23 months had a higher risk of overweight prevalence. This phenomenon has also been reported in other countries ^26^ ^,^ ^27^, which, in addition, have observed a more stable trend, with small reductions in the prevalence of overweight and obesity, especially in children and adolescents. However, the high prevalence of obesity in these age groups remains a concern ^25^ ^,^ ^28^ ^,^ ^29^ ^,^ ^30^.

In this study, we identified that among individuals in the < 12-month-old group, boys consistently had a higher obesity prevalence than girls, a difference that was not maintained in the 12-23-month-old group. These data are consistent with those obtained from Irish infants ^25^ ^,^ ^31^. Moreover, in Mexico, researchers have found statistically significant differences in overweight and obesity prevalence by sex in children and adolescents: the ENSANUTs show that, over the years, this prevalence is consistently higher in men ^32^.

In this study, the most relevant findings linked to region and indigeneity show that overweight and obesity prevalences in children under 24 months vary across the Mexican population, without presenting a specific behavior. In individuals with a high socioeconomic status, the prevalence was found to decrease from 2012 to 2020, possibly because this population has better access to medical care and healthier practices than populations from other socioeconomic status. These findings evidence the need to consider not only variations in socioeconomic status when developing methods to fight overweight and obesity, but also the environment and culture in which children and their families develop, since these variables could largely influence behaviors related to infant weight, such as eating habits and physical activity practices ^33^ ^,^ ^34^.

To effectively fight childhood obesity, which is a major problem, it is essential to develop prevention and intervention strategies focused on children’s first 1,000 days of life (pregnancy and first 24 months) and considering the main potentially modifiable risk factors shown by the evidence ^15^ ^,^ ^28^ ^,^ ^31^ ^,^ ^34^ ^,^ ^35^ ^,^ ^36^ ^,^ ^37^ ^,^ ^38^ ^,^ ^39^ ^,^ ^40^ ^,^ ^41^. Some strategies used during pregnancy are directed toward optimizing maternal health, achieving the recommended weight gain during pregnancy, and preparing women for successful breastfeeding. The strategies in the postnatal stage, which focus on the primary caregiver responsible for the child’s health, are centered on the promotion of adequate feeding practices (breastfeeding, complementary, and preceptive feeding) and the development of a healthy lifestyle for the child, which involves good eating habits, physical activity, and sleep quality.

In Mexico, national public policy recommendations that align with the above-mentioned strategies for the first 1,000 days of life have recently been formulated ^42^ ^,^ ^43^, but they are yet to be applied and evaluated. The application of these strategies to achieve healthy behaviors in future generations is considered a challenge for Mexico. Therefore, concerted public health efforts are needed in order to achieve healthy weight levels and nutritional goals, and to combat the epidemic of childhood obesity at an earlier age.

This study has several strengths. First, it was based on four ENSANUTs conducted in Mexico for almost 10 years. For these surveys, a team of trained health professionals collected data on children’s weight and height. Second, this study is the first to report the trend in the combined prevalence of overweight and obesity among children aged 0-23 months in Mexico over the past decade. However, this study also has some limitations: since data were obtained from four independent cross-sectional surveys, the trend in the combined prevalence of overweight and obesity could not be investigated longitudinally individually.

## Conclusion

In conclusion, the observed overweight + obesity prevalence in children aged < 24 months indicates that this condition is a public health problem in Mexico. This becomes even more evident when comparing this prevalence to the one found in countries with the same level of development as Mexico and located in same the region. Overweight and obesity puts children’s health, survival, and development at risk, which means that preventive efforts must be redoubled. Childhood obesity prevalence tends to stabilize or decrease in some high-income countries ^38^. This may have beneficial effects on the risk of obesity and its comorbidities throughout life in this age group, and gives us hope that the actions taken by these countries can also be taken by others, such as Mexico ^39^ ^,^ ^40^. It is essential to study the factors associated with obesity in the first 1,000 days of life in greater depth in Mexico, which will help design health actions and policies. In addition, it is important to constantly monitor the nutritional status of this age group in order to identify the groups with the highest overweight + obesity prevalence, which will allow the selection of priority groups for the implementation of preventive actions ^41^. Lastly, this implementation process and the effects of the various efforts to prevent poor nutrition should be evaluated.
